# Retraction: Tetrandrine Suppresses Lipopolysaccharide-Induced Microglial Activation by Inhibiting NF-κB and ERK Signaling Pathways in BV2 Cells

**DOI:** 10.1371/journal.pone.0224894

**Published:** 2019-10-31

**Authors:** The PLOS ONE Editors

After this article [1] was published, the following issues were raised about the western blot results reported in Figures 6 and 7:

- Bands in lanes 4 and 5 of the p-IKK blot in Figure 6A appear similar to one another and to lane 5 of the p-JNK panel in Figure 7A, when rotated 180 degrees.
- Similarities were noted between lane 1 of the p-IKK panel in Figure 6A and lane 1 of the p-JNK panel in Figure 7A, when rotated 180 degrees.
- There appear to be vertical discontinuities after lanes 1, 2, 3, 4, of the p-IKK panel in Figure 6A, after lanes 2 and 4 of the IKK panel in Figure 6A, after lanes 1, 2 and 4 of the β-actin panel in Figure 6A, and after lanes 2, 4 of the p-JNK panel of Figure 7A.
- In Figure 6A, there appears to be a horizontal discontinuity above the bands in the β-actin panel.
- In several panels there appear to be discontinuities or other irregularities in the background. In Figure 6A, p-p65 panel, there are areas below the bands in lanes 2–4 where the background pattern is discontinuous, i.e. areas of smooth background sharply abut areas of speckled background that are characteristic of other areas in the image. Similarly, the background around the band in lane 5 of the Figure 6A, p65 panel is smooth relative to the rest of the blot, as is the background above lanes 3, 5 of the p-JNK panel and above lanes 1–5 in the β-actin panel in Figure 7A.

The authors provided image files in support of the p65, p-p65, IKK, p-IKK, β -actin panels in Figure 6A, and for the p-JNK and β-actin panels of Figure 7A. The first author confirmed that in preparing figures they rearranged lanes and edited the images to remove some background noise.

For Figure 6A, the raw images supported the overall results reported in the figure, and clarified that lanes were rearranged when preparing the IKK, p-IKK, and β-actin panels. The first author noted that lane 4 data were duplicated in error in lane 5 in the p-IKK panel.

Per our editorial assessment, results in lanes 1–4 of the Figure 7A p-JNK panel do not match the raw image data provided for this experiment. Lane 1 of this panel appears to duplicate the data in Figure 6A, p-IKK, lane 1 and the raw image provided for the p-IKK experiment. The first author and corresponding author YZ confirmed this duplication and stated that this was due to an error in figure preparation. The first author also commented that they erased part of the image above the band shown in lane 3 of the Figure 7A p-JNK panel. Questions remain as to the data reported in lanes 2 and 4 of the p-JNK figure panel.

In light of the remaining concerns about the validity and reliability of the results reported in Figure 7A and the confirmed manipulation of background image data in the western blot panels of Figure 6 and 7, the *PLOS ONE* Editors retract this article.

The authors apologize for the figure concerns and commented that they had poor knowledge of *PLOS ONE*’s guidelines and of figure preparation best practice when they prepared the manuscript.

YD and YZ notified the journal that all authors agreed with the retraction, but are confident in the findings and stand by the published results. CZ confirmed their agreement, the other authors did not reply to the journal directly or could not be reached.
